# The genomic characterization of *Salmonella* Paratyphi A from an outbreak of enteric fever in Vadodara, India

**DOI:** 10.1099/mgen.0.000914

**Published:** 2023-01-06

**Authors:** Joana Pereira-Dias, Neelam Taneja, Jaspreet Mahindroo, Geeti Maheshwari, Padma J. Patel, Trang Nguyen Hoang Thu, Jacqui Keane, Zoe A. Dyson, Stephen Baker, Elli Mylona

**Affiliations:** ^1^​ University of Cambridge School of Clinical Medicine, Cambridge Biomedical Campus, Cambridge, UK; ^2^​ Department of Medicine, University of Cambridge School of Clinical Medicine, Cambridge Biomedical Campus, Cambridge, UK; ^3^​ Post Graduate Institute of Medical Education and Research, Department Medical Microbiology, Chandigarh, India; ^4^​ Department of Microbiology, Toprani Advanced Lab Systems, Vadodara, Gujarat, India; ^5^​ Oxford University Clinical Research Unit (OUCRU), Ho Chi Minh City, Vietnam; ^6^​ Department of Infection Biology, Faculty of Infectious and Tropical Diseases, London School of Hygiene & Tropical Medicine, London WC1E 7HT, UK; ^7^​ Department of Infectious Diseases, Central Clinical School, Monash University, Melbourne, Victoria 3004, Australia; ^8^​ Wellcome Sanger Institute, Wellcome Genome Campus, Hinxton, UK

**Keywords:** Enteric fever, India, Outbreak, Paratyphoid, QRDR

## Abstract

*
Salmonella enterica* Typhi (*S*. Typhi) and Paratyphi A (*S*. Paratyphi A) are the causative agents of enteric fever, a systemic human disease with a burden of 300 000 cases per year in India. The majority of enteric fever cases are associated with *S*. Typhi, resulting in a paucity of data regarding *S*. Paratyphi A, specifically with respect to genomic surveillance and antimicrobial resistance (AMR). Here, we exploited whole-genome sequencing (WGS) to identify *S*. Paratyphi A genotypes and AMR determinants associated with an outbreak of *S*. Paratyphi A in Vadodara, India, from December 2018 to December 2019. In total 117 *S*. Paratyphi A were isolated and genome sequenced, most were genotype 2.4.2 (72.6 % of all cases), which is the globally dominant genotype. The remainder were genotype 2.3 (25.6 %), while only two isolates belonged to genotype 2.4.1. A single base-pair mutation in *gyrA*, associated with reduced susceptibility to fluoroquinolones, was present in all of the outbreak isolates; with 74.35 % of isolates having a S83F substitution and the remainder having an S83Y substitution. Our surveillance study suggests that *S*. Paratyphi A is an emergent pathogen in South Asia, which may become increasingly relevant with the introduction of Vi conjugate vaccines.

## Data Summary

All short reads used in this study at European Nucleotide Archive (ENA) under study accessions of PRJEB54686 (ERS12453692 to ERS12453808).

We confirm that all supporting data, code and protocols have been provided within the article or through supplementary data files.

## Introduction

Enteric fever is a life-threatening systemic disease that can be caused by *Salmonella enterica* serovars Typhi (*S*. Typhi) and Paratyphi A (*S*. Paratyphi A), B and C. *S*. Paratyphi B and C are distantly related to both *S*. Paratyphi A and *S*. Typhi, and the incidence of these serovars is low [1, 2]. Poor water quality and inconsistent sanitation and hygiene conditions in some low middle income countries (LMICs) in South Asia, sub-Saharan Africa and Latin America make such location hot spots for many enteric infections, including enteric fever [3]. *S*. Typhi and *S*. Paratyphi A are the two pathogens responsible for the preponderance of enteric fever, accounting for ~80 % and ~20 % of the total burden of enteric fever globally, respectively [2]. Preventative measures, including improved water quality, have proven effective in reducing enteric fever in many endemic locations, but such approaches remain unachievable in many resource-poor settings [2, 4]. Consequently, although the annual global burden of the disease declined an estimated 44.6 % between 1990 and 2017, these organisms are still responsible for >200 000 deaths annually [2].

The implementation of typhoid conjugate vaccines (TCV) is likely to have a major impact on the incidence of enteric fever [5]. However, the protective antigen of TCV is the Vi capsular polysaccharide, which coats the surface of *S*. Typhi, but not *S*. Paratyphi A. Therefore, TCV is ineffective in preventing *S*. Paratyphi A disease [6], and there are currently no licensed vaccines against *S*. Paratyphi A [7]. Several surveillance studies conducted in Pakistan, India and China, have suggested that *S*. Paratyphi A infection rates are equivalent to, and in some cases greater than, the number of enteric fever cases caused by *S*. Typhi [8, 9]. Additionally, ineffective diagnostic tools may also contribute to an underestimation of the incidence and clinical relevance of *S*. Paratyphi A [10].

Multi-drug resistance (MDR; resistance to the first-line antimicrobials ampicillin, trimethoprim-sulfamethoxazole and chloramphenicol) was first identified in *S*. Typhi in the early 1990s [11]. Antimicrobial resistance (AMR) is not yet considered a major threat for *S*. Paratyphi A and MDR phenotypes are considered rare with a global prevalence of <0.5 %(73/29 731) among *S*. Paratyphi A between 1990–2018 [11]. However, a recent study described the presence of plasmid-mediated MDR in *S*. Paratyphi A isolated from patients in Bangladesh, China and India [12]. Additionally, resistance against nalidixic acid linked with reduced susceptibility against fluoroquinolones, is common in *S*. Typhi and has been reported in *S*. Paratyphi A [13]. In a comparable manner to *S*. Typhi and other Gram-negative organisms, mutations in the quinolone resistance determining region (QRDR) of *gyrA* and *parC* are associated with decreased fluoroquinolone susceptibility in *S*. Paratyphi A [14]. Consequently, the incidence of fluoroquinolone resistance in *S*. Paratyphi A has been increasing in Asia, suggesting that *S*. Paratyphi A may become a comparable AMR threat to *S*. Typhi [15–17]. AMR trends in *S*. Paratyphi A, the lack of licensed vaccines against this organism, and a slow improvement in WASH conditions, make tackling paratyphoid fever particularly challenging in LMICs.

The global prioritization of *S*. Typhi control, as determined by a higher incidence of disease (in comparison to *S*. Paratyphi A) has rendered *S*. Paratyphi A a neglected pathogen [18]. Therefore, compared to *S*. Typhi, there are limited contemporary epidemiological data and we do not have a well-developed genomic platform on which to study population structure and AMR patterns. With the aim of contributing new genomic data for *S*. Paratyphi A and evaluating AMR in this pathogen, we investigated a community outbreak of *S*. Paratyphi A in Vadodara, India in 2018–2019. We employed whole-genome sequencing (WGS) and utilized a recently developed typing scheme for *S*. Paratyphi A (Paratype) to describe the molecular epidemiology of this outbreak [19].

## Methods

### Sample collection, bacterial isolation and DNA extraction

A total of 117 *S*. Paratyphi A blood isolates (Table S1) were obtained from patients admitted to the hospital with enteric fever symptoms during an enteric fever outbreak in Vadodara, India in 2018 and 2019. The recruiting hospitals have no microbiology laboratory; therefore, samples for blood culture were sent to Toprani Advanced Lab systems for diagnosis purposes. Bacterial isolates were then transferred to PGIMER in Chandigargh and grown ON, 37 °C and DNA was extracted from liquid culture using Wizard genomic DNA purification kit (Promega Corporation, Madison, USA) as per the manufacturer’s protocol and stored at −20 °C.

### Antimicrobial susceptibility testing

Samples were routinely tested for susceptibility against ampicillin, cotrimoxazole, ceftriaxone, cefixime, chloramphenicol and azithromycin using the Kirby–Bauer disc diffusion susceptibility method and interpreted according to CLSI guidelines [20].

### Whole-genome sequencing and variant calling

Genomic DNA was quantified using Qubit 4.0 (ThermoFisher, MA, USA) and quality assessed using Tapestation (Agilent, CA, USA). Paired-end Illumina sequencing libraries were prepared using NEBNext Multiplex Oligos for Illumina (NEB, MA, USA), as per the manufacturer’s instructions. WGS was performed using the Illumina MiSeq (Illumina, CA, USA) generating 300 bp paired end reads. FASTQC (available at http://www.bioinformatics.babraham.ac.uk/projects/fastqc) was used to check raw read quality. A multi-alignment file was created by mapping the reads and SNP calling against the reference genome *S*. Paratyphi A AKU12601 (accession number: FM200053) using SMALT-v0.7.4.4 with default parameters. The multi alignment file was then screened for the presence of repetitive regions, prophages and recombinant regions using remove_blocks_from_aln.py (available at https://github.com/sanger- pathogens/remove_blocks_from_aln) and the coordinates file for these repetitive regions (available at https://github.com/katholt/typhoid; PARAREPEAT files), followed by a screen for loci containing high rates of recombination using Gubbins-v2.4.1. For a global context analysis, 848 publicly available *S*. Paratyphi A genomes, isolated between 1917–2019 across 35 countries (available in the European Nucleotide Archive – ENA) were also included in this analysis resulting in a total of 966 genomes (Table S2). From 848 global genomes, 606 were isolated from individuals returning to the UK post-international travel in 2016–2019, while the remaining genomes were retrieved from a previous study that performed global genetic analysis of *S*. Paratyphi A in circulation in 1917–2016 [21, 22].

### Phylogenetic analysis

Maximum-likelihood (ML) phylogenetic tree was created from the SNP alignment file using rAxML SSE3-v8.2.8, with 100 bootstraps (GTR+ Γ substitution model; GTRGAMMA in RAxML). A *S*. Typhi isolate was included as the outgroup for the tree rooting. Genotype was attributed based on Paratype genotyping tool (available at https://github.com/CHRF-Genomics/Paratype). Output files were visualized and annotated using FigTree v1.4.4 (available at https://github.com/rambaut/figtree) and ITOL (available at https://itol.embl.de/), respectively.

### Antimicrobial resistance genes and plasmid identification

SRST2-v0.2.0, together with ARGannot_r3.fasta and PlasmidFinder-v2.0.1 databases, was used to detect the presence of known AMR genes and plasmids, respectively. Point mutations in the *acrB* conferring resistance to azithromycin and in genes present in the quinolone resistance-determining region (QRDR) such as *gyrA* and *parC*, were identified using Genoparatyphi (available at https://github.com/zadyson/genoparatyphi). Raw sequence reads for the 117 study genomes were *de novo* assembled using Unicycler-v0.4.7 followed by Bandage-v0.8.1 visualization for the identification of potential novel plasmids. Circular replicons were further analysed in blastn at NCBI and any hypothetical proteins identified were further investigated using the webtool Phyre^2^ [23]. For the global collection analysis, a database including the circular replicon of interest, identified in within the 117 samples, was created and used in combination with SRST2-v0.2.0 to screen through the 848 global genomes. The presence of the circular replicon for the global collection samples was further confirmed with Unicycler-v0.4.7 followed by Bandage-v0.8.1 as per above.

## Results

### The population structure of *S*. Paratyphi A from Vadodara

**Fig. 1.**
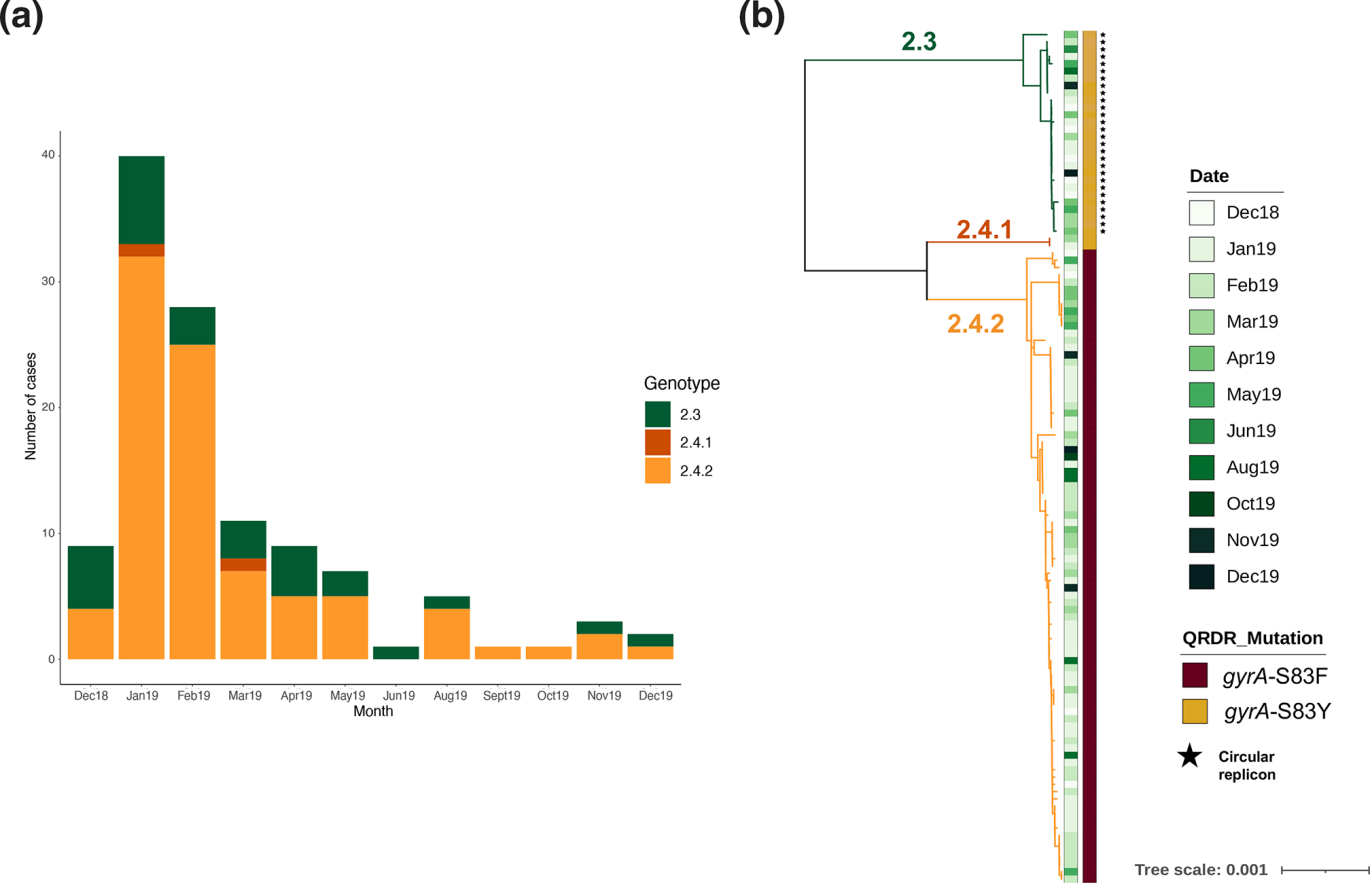
The population structure and genotype distribution of *S*. Paratyphi A isolates in a Vadodara outbreak. (a) Bar graph showing the genotype distribution of *S*. Paratyphi A across time. (b) SNP-based maximum-likelihood phylogenetic tree of 117 *S*. Paratyphi A isolates from Vadodara. Different branch colours highlight the genotypes identified. From left to right, annotations show the month of isolation, QRDR mutation (*gyrA*) and the presence of a plasmid represented with a black star.

A total of 117 *S*. Paratyphi A cases were identified within a radius of ~100 km from Vadodara city in Gujarat province along the western coast of India between December 2018 and December 2019. The peak of the outbreak was between January and February 2019, where the number of cases was more than half of the total amount of cases (69/117; 58.9 %) over the study period (Fig. 1a). All 117 patients admitted to the city hospital had symptoms of enteric fever including nausea, abdominal discomfort and fever. *S*. Paratyphi A was isolated from the blood of these patients, the median age of the patients was 10 years (range 1–73; IQR 13), and 61.5 %(72/117) were male. These numbers were considered higher than normal over this year-long period, and an outbreak was suspected, the organisms were subject to WGS to investigate the nature of the outbreak.

Phylogenetic analyses of the *S*. Paratyphi A genomes from this Vadodara outbreak and a global collection were performed. The total alignment length for the 117 Vadodara *S*. Paratyphi A genomes and for the 848 global collection genomes was 4 794 508 bp, which contained 442 and 7071 chromosomal SNPs, respectively. The resulting phylogeny of the 117 genome sequences from Vadodara revealed that the isolates could be divided into three major genotypes, suggesting that it was not a single organism/source outbreak (Fig. 1b). The majority (87/117; 74.35 %) of isolates were assigned to subclade 2.4, previously known as lineage A, and the remaining were assigned to genotype 2.3, formerly known as lineage C. Subclade 2.4 could be further divided into genotypes 2.4.1 (2/87; 2.3 %) and 2.4.2 (85/87; 97.7 %). Temporal data (month of isolation) did not suggest a replacement of genotypes over time, neither a cluster of genotypes over a particular time frame (Fig. 1a). Due to a lack of precise geolocation data (i.e. no postcode or GPS data were available), no conclusions could be drawn regarding the spatial clustering, geographic restriction and/or local transmission of these specific genotypes (data not shown).

**Fig. 2.**
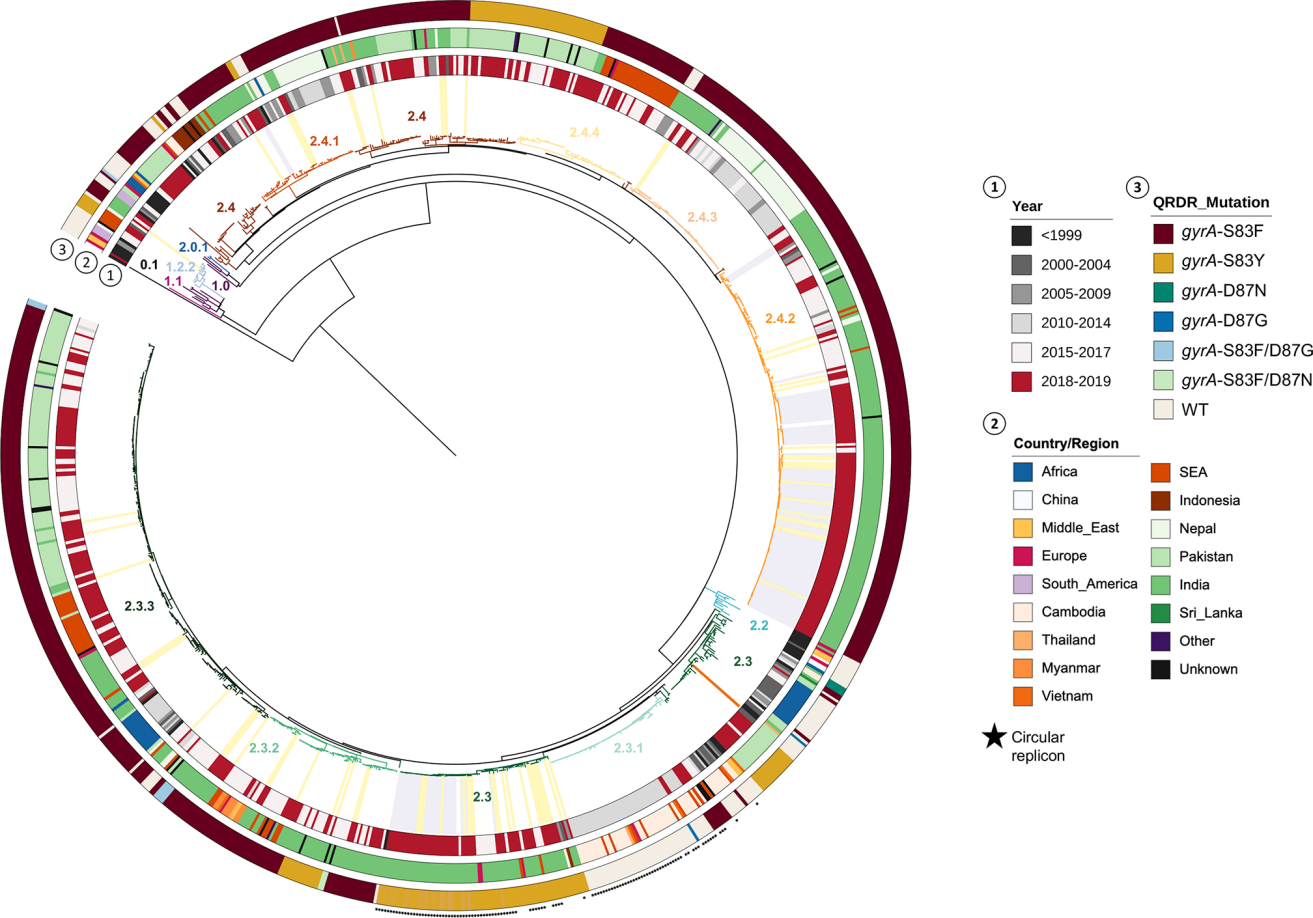
Phylogenetic representation of *S*. Paratyphi A isolates from Vadodara in a global context SNP-based maximum-likelihood phylogenetic tree of 966 global *S*. Paratyphi A isolates including the 117 Vadodara isolates. Different branch colours highlight identified genotypes. Clades for the isolates from the Vadodara outbreak are highlighted in yellow, 71 genomes isolated in the same time period from UK travellers to India are highlighted in grey and the clade for the reference genome *S*. Paratyphi A AKU12601 is highlighted in orange. From the inside, ring 1 and 2 correspond to the year and country of isolation, respectively, and ring 3 shows the QRDR mutations identified.

A phylogenetic analysis of global isolates (Fig. 2) revealed that the genotypes 2.4.1 from Vadodara were closely related to previously identified organisms of the same genotype originating from India, Nepal and Cambodia (median distance of 119.5 SNP; minimum=13, maximum=224). Similarly, the genotype 2.4.2 had a median distance of 16 SNPs (minimum=0, maximum=224) from other 2.4.2 organisms isolated in India, Nepal and Southeast Asia. Notably, the genotype 2.3 *S*. Paratyphi A from this present study were closely related to isolates from India, Nepal, Southeast Asia (including Indonesia) and Europe with a median SNP distance of 25 (minimum=0, maximum=234). These data suggest the geographical circulation of these three genotypes amongst neighbouring countries between Asia and Southeast Asia, as well as the potential for repeated introduction of both these major *S*. Paratyphi A genotypes into India. Moreover, isolates from travellers returning to the UK from India in 2019 (Fig. 2 – grey highlight) cluster with study isolates from Vadodara (Fig. 2 – yellow highlight) belonging to genotype 2.3 with a median SNP distance of 25 SNPs (minimum=0, maximum=220), 2.4.1 with a median SNP distance of 122.5 SNPs (minimum=23, maximum=212) and 2.3 with a median SNP distance of 13 SNPs (minimum=0, maximum=220). Notably, out of the 71 travellers isolates contemporary to this study, 12.6 % (9/71; *n*=4 for genotype 2.3 and *n*=5 for genotype 2.4.2) had an identical SNP composition to at least one of the Vadodara isolates, suggesting a possible transmission of these genotypes to Western countries, through travellers returning from India.

### AMR gene profile in the Vadodara *S*. Paratyphi A collection

Antimicrobial susceptibility was performed by disc diffusion; all *S*. Paratyphi A isolates were found to be susceptible to ampicillin, cotrimoxazole, ceftriaxone, cefixime, chloramphenicol and azithromycin but resistant to ciprofloxacin. These data were supported by a lack of detectable AMR-associated genes across this 117 genome collection. Conversely, all isolates possessed non-synonymous mutations at position 83 in *gyrA*, which is associated with reduced susceptibility to fluroquinolones. No additional QRDR mutations in *gyrA* or *parC* were identified [14]. At position 83 of *gyrA*, 74.4 % (87/117) of sequences contained substitutions from serine (S) to phenylalanine (F) and 25.6 %(30/117) to tyrosine (Y). These data corroborate previous findings where mutations at position 83 in *gyrA* of *S*. Paratyphi A were determined to be being the most commonly associated with reduced quinolone susceptibility in *S*. Paratyphi A in India [14]. The sequences were additionally screened for the presence of a single base-pair mutation in position 717 of the AcrB efflux pump, which has been linked to an increased macrolide resistance in *Salmonella* serovars Typhi and Paratyphi A from India, Bangladesh and Nepal [24]; no such mutation was identified in the Vadodara outbreak *S*. Paratyphi A sequences.

### Plasmid profiles in *S*. Paratyphi A from Vadodara

Lastly, we aimed to identify plasmid-associated sequences within this contemporary collection of *S*. Paratyphi A genomes. Using PlasmidFinder database, we found no known plasmid replicons in this collection. However, a plasmid prediction, using Bandage [25], revealed that 25 isolates carried an identical 2 734 bp novel circular replicon; these isolates were all within genotype 2.3 (Fig. 1). Additionally, the same replicon was also identified in 46 genomes within the global collection belonging within genotype 2.3.1. *In silico* analysis determined 97.26 % sequence identity between this circular replicon and the 2 573 bp long *E. coli* plasmid p5_025970 (accession number: CP036184.1), which encodes a single hypothetical protein of unknown function. Further *in silico* structural analysis indicated that this gene is likely to encode for a putative DNA-binding protein (95.4 % confidence).

## Discussion

In the absence of improvements in WASH conditions in many LMICs, vaccines are the major mechanism to reduce the incidence of enteric fever [26]. TCV is now being rolled out in many countries in Asia and Africa, which protects against *S*. Typhi; however, people in LMICs remain susceptible to *S*. Paratyphi A infections, against which there is currently no available prophylactic measure. Such a scenario, in combination with an increase in AMR in *S*. Typhi and the potential for the emergence of resistance in *S*. Paratyphi A, makes it critical to monitor circulating *S*. Paratyphi A genotypes, the locations in which they cause disease, and the burden of each genotype, particularly to record baseline data on *S*. Paratyphi A post-TCV implementation. Therefore, the use of WGS and SNP analysis to study 117 *S*. Paratyphi A genomes, isolated in Vadodara, India, from December 2018 to December 2019 represents a snapshot of *S*. Paratyphi A disease in India. In accordance with Swachh Survekshan, a survey for cleanliness, hygiene and sanitation across Indian cities and villages, Vadodara was consistently in the top ten cleanest cities from 2016, except for 2018 and 2019. In these years, Vadodara went down in ranking to positions 44th and 78th, respectively, corresponding to an upsurge in *S*. Paratyphi A disease, suggesting that the increase of *S*. Paratyphi A isolation is associated with environmental contamination leading to a long-cycle of transmission [27].

Phylogenetic analyses revealed that out of the three genotypes identified (2.3, 2.4.1 and 2.4.2) most isolates belonged to genotype 2.4.2 (72.6 %). These findings are consistent with the study by Tanmoy *et al*. (2021) who identified 2.4.2 as being the most prevalent *S*. Paratyphi A genotype in India, accounting for 22 % of the globally identified cases [28]. *S*. Paratyphi A has a highly conserved genome with great deficiency in the ability to acquire new genes from other species [29]. This feature and the low rate of SNP accumulation of 1.94×10^−7^ SNP per nucleotide per year (< 1 SNP per genome per year) in *S*. Paratyphi A [9], suggests that this upsurge in cases in Vadodara was not caused by a single clone but by multiple *S*. Paratyphi A genotypes. Notably, it has previously been reported that genotype 2.4.1 accounted for 19 % of enteric fever cases caused by *S*. Paratyphi A in India, whereas genotype 2.3 was responsible for 16 % [28]. However, in the present study, genotype 2.3 was the second most identified. Moreover, considering that genotype 2.4.1 was isolated only twice during the period of investigation (1.7 % of cases), in January and March 2019, and was also present across every year interval since before 1999, shown by the global analysis of this study, it is likely that these isolates are a component of endemic *S*. Paratyphi A in the region. Unfortunately, due to lack of associated clinical/epidemiological data, we cannot speculate on the introduction and/or transmission of these genotypes. Interestingly, the observed peak of *S*. Paratyphi A infections in Vadodara in the beginning of 2019 was coincident with a peak of paratyphoid cases reported in the UK in travellers returning from India in the same year [21]. This increase of paratyphoid fever cases in travellers returning from India over the same time frame, with 12.6 % of the isolates being 100 % identical to Vadodara isolates, suggests that the high number of cases verified in Vadodara was probably not only justified by an increase of cases restricted to this region of Gujarat district but reflected an overall increase in *S*. Paratyphi A infections in the country, in particular genotype 2.3 and 2.4.2.

Enteric fever has been historically treated with ampicillin, trimethoprim-sulfamethoxazole or chloramphenicol. The emergence of MDR *S*. Typhi associated with the presence of IncHI plasmid promoted a change of enteric fever treatment from these first-line antimicrobials to the use of fluoroquinolones (ciprofloxacin, ofloxacin and levofloxacin) as empirical treatment of enteric fever [30]. Although the rates of AMR are not as high in *S*. Paratyphi A in comparison to *S*. Typhi, an increase in *S*. Paratyphi A resistance to fluoroquinolones associated with QRDR *gyrA* and *parC* has been reported [15, 31]. Indeed, phenotypic and genotypic data demonstrated that the entire collection of organisms from Vadodara exhibited decreased susceptibility to ciprofloxacin. The fact that the *gyrA-*S83F mutation was the most prevalent among the Vadodara isolates when compared to *gyrA*-S83Y, corroborates previous findings that identified S83F mutation as being the most common in *S*. Paratyphi A [32]. Some recent studies have identified azithromycin resistance in *S*. Typhi and *S*. Paratyphi A, which is associated with a lone SNP in the *acrB*; this mutation was not identified here [24, 33]. Notably, no AMR-associated plasmid sequences were identified; however, a small circular body of 2 734 bp was identified in genotype 2.3 isolates from Vadodara and across most of the global collection samples belonging to genotype 2.3.1. This plasmid does not seem to be spreading across genotypes circulating in the same time period, but was restricted to two genotypes and warrants further investigation.

Overall, these data provide new insights into the population of *S*. Paratyphi A causing an enteric fever outbreak in the Indian city of Vadodara and we note a high prevalence of reduced susceptibility to fluoroquinolones. To our knowledge, the presented study is the first to describe an outbreak of *S*. Paratyphi A in India that has been characterized by WGS and a robust genotyping scheme. Consequently, to extend the existing *S*. Paratyphi A genotyping framework in a comparable manner to *S*. Typhi, we encourage that comparable studies using genomics surveillance are performed in locations with endemic enteric fever caused by *S*. Paratyphi A.

## Supplementary Data

Supplementary material 1
